# Frontal Alpha Asymmetry, a Potential Biomarker for the Effect of Neuromodulation on Brain’s Affective Circuitry—Preliminary Evidence from a Deep Brain Stimulation Study

**DOI:** 10.3389/fnhum.2017.00584

**Published:** 2017-12-04

**Authors:** Lihua Sun, Jari Peräkylä, Kaisa M. Hartikainen

**Affiliations:** ^1^Behavioral Neurology Research Unit, Tampere University Hospital, Tampere, Finland; ^2^Turku PET Center, University of Turku, Turku, Finland; ^3^Faculty of Medicine and Life Sciences, University of Tampere, Tampere, Finland

**Keywords:** deep brain stimulation, neuromodulation, frontal alpha asymmetry, emotion, biomarker, epilepsy, anterior thalamic nuclei, anterior nucleus of thalamus

## Abstract

Neuromodulation techniques targeting limbic circuits can be used to treat refractory psychiatric or neurological disorders. However, objective measure for the impact of neuromodulation on affective brain circuits is lacking. Deep brain stimulation at a key node of the limbic circuit, the anterior thalamic nuclei (ANT-DBS), is used to treat refractory epilepsy. While effective in reducing seizures, patients have reported subjective depressive symptoms as a side effect. In line with potential vulnerability to depression, we have previously shown ANT-DBS to increase attention allocation to threat evidenced by behavior and brain physiology. Rightward frontal alpha asymmetry with greater right hemispheric activation is thought to reflect brain physiology linked with depression and anxiety. To that end, we investigated whether high-frequency electric stimulation at ANT influences frontal alpha asymmetry. Furthermore, we explored the impact of DBS on emotional modulation of frontal alpha asymmetry and whether it is linked with emotional modulation of response speed. Electrical stimulation at ANT led to an increased rightward frontal alpha asymmetry compared to situations where stimulation was off (*F*_(1,12)_ = 14.09, *p* = 0.003) or the thalamic control location was stimulated (*F*_(1,12)_ = 10.19, *p* = 0.008), along with prolonged reaction times in the context of emotional distractors (*F*_(1,7)_ = 16.66, *p* = 0.005). The change was specifically driven by increased activity in the right hemisphere. Furthermore, we found a correlation between the emotional modulation of frontal alpha asymmetry and emotional interference of response speed due to ANT stimulation (*r* = 0.78, *p* = 0.02). In conclusion, DBS at ANT increased relative right hemispheric activity and this was linked with emotional modulation of behavior. Previous studies have linked frontal alpha asymmetry with emotion related symptoms and furthermore, Vagus Nerve Stimulation (VNS) has been shown to modulate alpha asymmetry. Thus, in the light of the previous literature and the current findings, we suggest that frontal alpha asymmetry along with emotional interference of response speed might be a feasible biomarker for the effects of neuromodulation on brain’s affective circuitry in general.

## Introduction

Neuromodulation techniques are increasingly used for treating severe neurological and psychiatric disorders. For instance, deep brain stimulation (DBS) at the anterior thalamic nuclei (ANT) is used for treating refractory epilepsy with large scale clinical studies indicating significant benefits in reducing seizure frequency (Fisher et al., 2010) and demonstrating sustained benefits within a 5-year follow-up period (Salanova et al., 2015). Along with treatment effects ANT-DBS has been linked with adverse mood related effects such as subjective depression-related symptoms (Fisher et al., 2010). Affective adverse effects are not surprising considering the treatment is targeting ANT, which is a key node in the limbic circuitry. To that end there is a call for objective biomarkers for the impact of neuromodulation on affective brain functions. Biomarkers which allow guiding neuromodulation treatments, for example parameter selection towards the optimal treatment effect and minimal cognitive and affective side effects, have great clinical significance. The importance of such biomarkers is emphasized especially when neuromodulation is used to treat emotional dysfunctions, for example depression.

EEG provides a feasible method for assessing online alterations in human brain physiology and it is frequently used in both clinical and research settings. Thus, it is not surprising that different EEG measures have been suggested as potential biomarkers for variety of brain disorders and for the impact of neuromodulation on brain functions (Coan and Allen, 2004; Thibodeau et al., 2006; Grimshaw et al., 2014; Sun et al., 2017), as well as for guiding DBS electrode targeting using cortical responses to deep brain stimuli providing information about optimal electrode locations (Valentín et al., 2013). Measures quantifying EEG frequency information such as theta concordance have been suggested to have utility as a potential biomarker for treatment response in DBS at subcallosal cingulate for treatment-resistant depression (Broadway et al., 2012). The profile of asymmetric EEG activity of different frequency bands has been suggested to reflect potential response to DBS at subgenual cingulate cortex (Quraan et al., 2014). The suggested measures have shown potential in distinguishing responders from non-responders, which is important when selecting patients who will most likely benefit from an invasive neuromodulation treatment. It is equally important to have biomarkers for the immediate impact of neuromodulation on affective functions. Such biomarkers will allow personally tailored neuromodulation parameter adjustments and provide immediate feedback about the impact of chosen parameters on the affective brain networks and affective functions enabling online optimization of the stimulation parameters. In the current clinical practice parameters are adjusted over the course of several months of follow up based on subjective reports rather than objective biomarkers reflecting the impact of neuromodulation on brain’s affective circuits.

In search for online biomarkers for neuromodulation techniques, we have previously shown that Vagus Nerve Stimulation (VNS), a neuromodulation technique used for the treatment of refractory epilepsy and depression, has immediate modulatory effect on brain’s frontal alpha asymmetry (Sun et al., 2017). We compared the frontal asymmetry score when cyclic VNS was turned on vs. when it was turned off and found that VNS led to increased rightward frontal alpha asymmetry in the context of emotional distractors. In the context of neutral distractors, however, VNS had no effect on frontal alpha asymmetry. This finding revealed an immediate modulatory effect of VNS on emotion-related brain responses and highlighted the potential utility of frontal alpha asymmetry as an online biomarker of the impact of neuromodulation on brain’s affective functions.

Frontal EEG alpha asymmetry indicating increased relative right hemispheric activity has been frequently linked with depression and anxiety (Coan and Allen, 2004; Thibodeau et al., 2006) even though the recent meta-analysis suggests quite variable and unreliable effects (van der Vinne et al., 2017). Rightward frontal EEG alpha band asymmetry, i.e., relatively increased right hemisphere activation, has been reported in both currently and previously depressed subjects compared to never depressed subjects (Henriques and Davidson, 1990, 1991; Gotlib, 1998; Stewart et al., 2011). The same pattern of frontal asymmetric activation is found in infants of mothers with depression (Field and Diego, 2008; Wen et al., 2017). Also, rightward shift in frontal asymmetry is linked with current anxiety and it can potentially predict future anxiety (Tomarken and Davidson, 1994; Blackhart et al., 2006; Mathersul et al., 2008).

Both resting state and task-related frontal alpha asymmetry have been studied. Resting state frontal alpha asymmetry is recorded when subjects are not engaged in behavioral tasks. In contrast, behavioral tasks require attentional engagement and tasks with emotional elements may promote asymmetric activation of the brain hemispheres. Therefore, resting state frontal alpha asymmetry indexes hemispheric brain activity at rest, while task-related alpha asymmetry indexes brain activity during a behavioral task (Coan and Allen, 2004). It has been suggested that task-related frontal alpha asymmetry may be a more powerful indicator of predisposition towards psychopathology than resting state alpha asymmetry, possibly due to elimination of the uncontrolled variance of each subject at resting state (Coan et al., 2006; Stewart et al., 2014). Also, given the high demand of attention in behavioral tasks, the task-related frontal alpha asymmetry may better link brain’s emotional-processing-related asymmetric activation with cognitive processes, such as attention and executive control (Grimshaw et al., 2014).

Previously, we have studied the effects of ANT-DBS on human attention and executive functions with objective measures using a computer-based Executive-RT test (Hartikainen et al., 2010, 2014). The experimental test has been developed based on our previous neurocognitive studies on healthy subjects and patients with frontal lesions while employing reaction time and event-related potential measures (Hartikainen et al., 2000, 2007; Hartikainen and Knight, 2003). The Executive-RT test requires multiple cognitive processes to be engaged simultaneously and has been shown to detect subtle changes in attention and emotion-attention interaction due to different types of neuromodulation techniques (Hartikainen et al., 2014; Sun et al., 2015, 2016, 2017; Sun, 2016; Peräkylä et al., 2017), aortic valve replacement surgery (Liimatainen et al., 2016), mild brain traumatic injury (Mäki-Marttunen et al., 2015) and orbitofrontal lesions (Mäki-Marttunen et al., 2017). Using this test, we found that ANT-DBS led to increased emotional interference, evidenced by increased reaction times in the context of emotional distractors and increased attention allocation to threat-related stimuli, evidenced by increased centro-parietal N2-P3 brain potentials (Hartikainen et al., 2014; Sun et al., 2015).

In the current study, we investigated whether ANT-DBS promotes immediate changes in asymmetric frontal activation and whether these changes are associated with processing of negative emotions. Given our previous findings with ANT-DBS increasing attentional allocation to emotionally negative stimuli (Hartikainen et al., 2014; Sun et al., 2015), reports where ANT-DBS has been linked with subjective depressive symptoms (Fisher et al., 2010) and meta-analysis linking frontal rightward alpha asymmetry activity with depression (Coan and Allen, 2004; Thibodeau et al., 2006), we expected ANT-DBS to lead to increased rightward frontal alpha asymmetry, i.e., relatively increased right hemisphere activation.

## Materials and Methods

### Subjects

Thirteen patients with ANT-DBS treatment for refractory epilepsy participated in this study. Twelve subjects included in our previous publications (Hartikainen et al., 2014) were complemented with one new subject. Subjects had frontal (*n* = 4), temporal (*n* = 1), occipital (*n* = 2) and multifocal (*n* = 6) epilepsies, and the main etiologies were cortical dysplasia (*n* = 5) and encephalitis (*n* = 5). Three subjects had unknown etiology. Six subjects had pathological findings in Magnetic Resonance Imaging (MRI) and seven had normal MRIs. All subjects were using one to four anti-epileptic medications. The wide heterogeneity of the patients with variety of factors potentially contributing to behavior and EEG was accounted for by a within-subject study design. The study was approved by the Regional Review Board at Tampere University Hospital, Tampere, Finland. All subjects provided their written consent and voluntarily participated in the study according to the guidelines set forth in the Declaration of Helsinki governing the treatment of human subjects.

### Executive RT Test

Patients performed the Executive-RT test (Hartikainen et al., 2010), a visual attention task with emotional distractors, while their scalp EEG was recorded. During the test, patients sat one meter away from a 21″ computer screen. The behavioral paradigm was presented using Presentation software (Neurobehavioral System Inc., Berkeley, CA, USA). The whole test consisted of two sessions, each session consisting of 16 blocks and each block consisting of 64 two-second trials. The onset of each trial was a triangle pointing either up or down and lasting 150 ms. After the triangle, there was a lapse of 150 ms before a Go/NoGo signal. Go/NoGo signal was a traffic light in either green or red color presented for 150 ms. The color of the traffic light indicated whether subject should respond or not, i.e., a Go or a NoGo trial. In a Go trial, subjects were supposed to press one button using their index (down button) or middle finger (up button) to indicate the orientation of the previously presented triangle. In a NoGo case, patients should not press any buttons. The meaning of the traffic light color changed from block to block so that in half of the blocks green indicated Go and red NoGo signal and in another half the meaning was inverted so that red indicated Go and green indicated NoGo. In the middle circle of the traffic light there was a schematic line drawing distractor, which was either a negatively valenced shape of a spider or emotionally neutral control distractor composed of the exactly same graphical components and resembling a flower.

Behavioral outcomes of the test include reaction times and different types of errors. Here we report only emotion related behavioral findings. Analysis of errors did not give emotion-related significance, see Peräkylä et al. (2017).

### Stimulation Protocol

All patients received ANT-DBS treatment for refractory epilepsy with DBS leads bilaterally in the anterior thalami. “ANT location” refers here to electrode locations inside ANT or immediately adjacent to ANT with two subjects having “ANT location” slightly outside of ANT (see also Sun et al., 2015). One subject used left hand for performing the behavioral test.

During the experiment, bipolar stimulation was used to allow better targeting of ANT. The DBS lead has four electrodes out of which we used two adjacent electrodes closest to ANT when ANT was stimulated, with one as negative contact and the other as positive contact. Continuous electrical stimulation with a constant 5 mA current, 140 Hz frequency and 90 μs pulse width was given when stimulation was ON. When stimulation was OFF, no current was delivered. When the control location outside ANT was stimulated, the two farthest contacts from ANT were chosen as active contacts. The control location was referred to as “outside ANT location (OA)” in our previous study (Hartikainen et al., 2014). The control location contacts were typically in adjoining inferior structure to ANT, e.g., mediodorsal nucleus.

**Figure 1:**
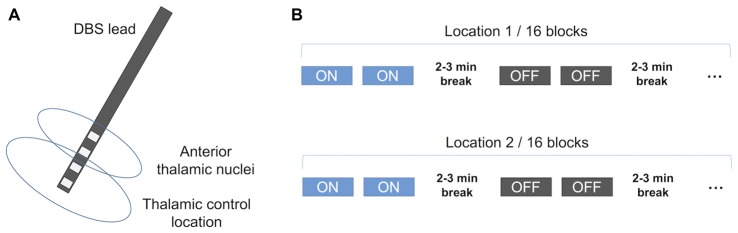
Schematic illustration of the stimulation protocol. **(A)** The DBS lead and the stimulated sites. Each lead has four potentially active contacts. In bipolar stimulation, two contacts were active with one cathode and the other anode. Typically, superior contact pair was in the anterior thalamic nuclei (ANT) and inferior contact pair was considered to be outside ANT assigned as the thalamic control location. **(B)** The whole experiment for each subject included 32 blocks of testing. In the first 16 blocks, either the ANT or the control location was stimulated, or stimulation was turned off. In the second 16 blocks, the other location was stimulated, or stimulation was turned off. The order of stimulated sites was counterbalanced. DBS, deep brain stimulation. This figure is adapted from Peräkylä et al. (2017).

The experiment consisted of two sessions (see Figure 1). In one session stimulation was alternatively ON or OFF at ANT, and in the other session stimulation was alternatively ON or OFF at control location. The starting order of the sessions was counter balanced between subjects. Each status, e.g., ANT ON, lasted for 5–6 min to allow for completing two blocks of testing. Therefore, eight blocks of tests were done with ANT ON status, eight blocks of tests were with control location ON status, and 16 blocks were done with OFF status (i.e., ANT OFF and control location OFF).

### Frontal Alpha Asymmetry

EEG signal was recorded using the 64-channel Ag/AgCl electrodes and the QuickAmp EEG amplifier (Gilching, Germany). EEG signal was processed offline using the Brain Analyzer 2 software (Brain Products GmbH, Germany). First, signal was down sampled to 250 Hz. Ocular movements were corrected using the ICA ocular correction function of the Analyzer 2 software, where signals are decomposed into independent components with the extended Infomax algorithm and one to two components corresponding eye movements were rejected. Then the signal was re-referenced to the central EEG electrode Cz and filtrated with bandpass filter at 3–30 Hz. EEG signal was segmented into 2-s segments, with each starting from the onset of the trial, i.e., the triangle. EEG segments with amplitudes exceeding ±80 μV were removed. The power spectrum (μV^2^/Hz) of all segments was calculated using the Fast Fourier transformation and averaged for each condition. Spectral power (μV^2^) at the alpha range (8–13 Hz) was exported. Finally, alpha power of EEG electrodes F3 and F4 was log-transformed and the asymmetry score of frontal alpha was calculated by subtracting the value at F3 from the value at F4 (log-transformed spectral power at F4—log-transformed spectral power a F3).

### Statistics

Frontal alpha asymmetry and hemispheric alpha activity were analyzed for the whole group (*n* = 13) and for a subgroup of subjects who completed the task with good performance (*n* = 8). A subject was considered a good performer if subject’s total error rate was below 15% (Hartikainen et al., 2014).

Frontal alpha asymmetry scores and reaction times were analyzed using repeated measures analysis of variance using Emotion (emotional, neutral) and Stimulation (ANT ON, OFF, control location ON) as factors. Hemispheric alpha activity was analyzed using repeated measures analysis of variance using Emotion, Stimulation and Hemisphere (left, right) as factors. In subgroup analysis, the factor Stimulation has two levels (ANT ON and OFF) in order to control for multiple comparison. Before analysis, distribution of the data was tested for normality and transformed by subtracting subject mean from subject’s values if necessary. Significant interaction effects were followed by *post hoc* analysis. Bonferroni-adjusted significance criteria *p* = 0.017 was used to correct for multiple comparison. All statistical findings were confirmed with non-parametric statistical approach using Wilcoxon signed rank test as detailed in our previous study (Sun et al., 2015).

#### Correlation Analysis

We studied how ANT-DBS modulates emotional processing by analyzing changes ANT-DBS causes in emotion-related alpha asymmetry and emotion-related reaction times. Subtracting alpha asymmetry scores in the context of neutral stimuli from alpha asymmetry scores in the context of emotion-related stimuli gives an index for the impact of emotional stimuli on alpha asymmetry, with other factors contributing to the alpha asymmetry scores controlled for by subtraction. Similarly, subtracting reaction times in context of neutral stimuli from RTs in context of emotion-related stimuli gives an index indicating the impact of emotional stimuli on response speed. After deriving these measures for each stimulation status (i.e., ANT ON and OFF) separately, we subtracted obtained values when stimulation was OFF from the values when ANT stimulation was ON, resulting in net impact indices of ANT-DBS and emotion-related stimuli on frontal alpha asymmetry scores and reaction times.

After deriving the net impact indices for frontal alpha asymmetry and reaction times, we conducted a correlation analysis where we correlated changes caused by ANT stimulation on reaction times and alpha asymmetry linked to emotion. This analysis was done only for good performers. Because reaction time and alpha asymmetry scores varies hugely between subjects, they were transformed to z-score before the analysis and all calculations were done using z-scores.

All statistical analysis was done in R statistics (version 3.2.3). Repeated measure analysis of variance was done using the ez package (Lawrence, 2013).

## Results

### Frontal Alpha Asymmetry and Hemispheric Activity, Whole Group

**Figure 2:**
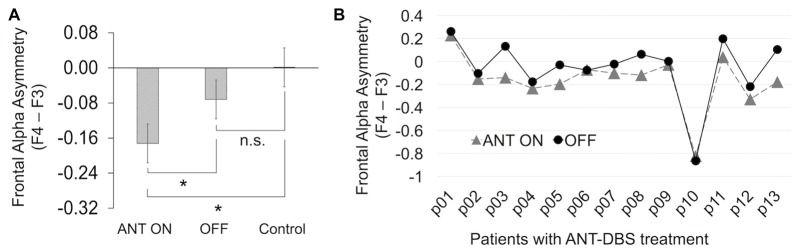
ANT-DBS led to increased rightward frontal alpha asymmetry.** (A)** When ANT was stimulated, enhanced rightward frontal asymmetry (smaller asymmetry score) was found in comparison to situations when stimulation was OFF and when thalamic control location (Control) was stimulated. *Statistically significant; n.s., no significance. Error bars represent Fisher’s least significant difference. **(B)** Individual changes in the frontal alpha asymmetry when ANT was stimulated and when it was not.

Analysis of frontal alpha asymmetry scores resulted in a main effect of Stimulation (*F*_(2,24)_ = 10.01, *p* = 0.0007, $\eta_{\text{G}}^{2}$ = 0.074). To compare the difference between stimulation status, a pairwise analysis was done. Stimulation of ANT led to increased rightward frontal alpha asymmetry (i.e., relatively less alpha power on the right) compared to OFF (*F*_(1,12)_ = 14.09, *p* = 0.003, $\eta_{\text{G}}^{2}$ = 0.085) and ON at control location (*F*_(1,12)_ = 10.19, *p* = 0.008, $\eta_{\text{G}}^{2}$ = 0.104), Figure 2A. Under the Bonferroni-adjusted significance criteria, there was an opposite trend when control location was stimulated with decreased rightward frontal alpha asymmetry compared to OFF situations (*F*_(1,12)_ = 6.61, *p* = 0.025, $\eta_{\text{G}}^{2}$ = 0.021). Subject specific effects of ANT-DBS on frontal alpha asymmetry are illustrated in Figure 2B.

Analysis of the hemispheric alpha activity resulted in an interaction effect between Stimulation and Hemisphere (*F*_(2,24)_ = 14.09, *p* = 0.0007, $\eta_{\text{G}}^{2}$ = 0.041). Under the Bonferroni-adjusted significance criteria, there was a trend towards increased right hemispheric activity when ANT was stimulated (*F*_(1,12)_ = 5.89, *p* = 0.03, $\eta_{\text{G}}^{2}$ = 0.14), with left 1.32 ± 1.11 μV and right 1.16 ± 1.07 μV. This tendency did not exist when control location was stimulated (*F*_(1,12)_ = 0.01, *p* = 0.75, $\eta_{\text{G}}^{2}$ = 0.004) or when stimulation was OFF (*F*_(1,12)_ = 0.53, *p* = 0.48, $\eta_{\text{G}}^{2}$ = 0.039).

### Frontal Alpha Asymmetry, Hemispheric Activity and Emotional Modulation of Behavior, Good Performers

To increase statistical power and control for multiple comparison, subgroup analysis was done only for ANT ON and OFF situations. Specifically, instead of using a three-level factor Stimulation (ANT ON, OFF and control location ON), we used a two-level factor Stimulation (ANT ON and OFF) in the following statistical analysis.

Analysis of the frontal alpha asymmetry scores for good performers resulted in comparable results to whole group analysis, i.e., a main effect of Stimulation (*F*_(1,7)_ = 9.75, *p* = 0.017, $\eta_{\text{G}}^{2}$ = 0.38). Additionally, interaction effect between Stimulation and Emotion was marginally significant (*F*_(1,7)_ = 4.94, *p* = 0.06, $\eta_{\text{G}}^{2}$ = 0.003).

**Figure 3:**
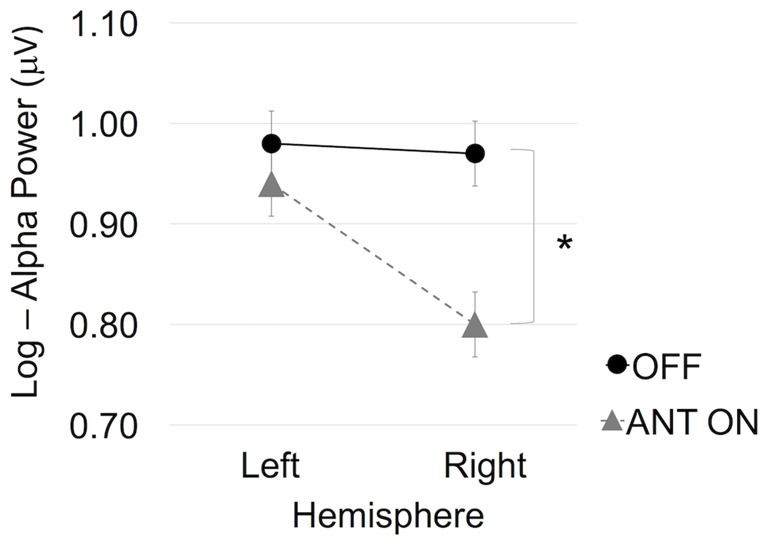
ANT stimulation led to significantly increased right hemispheric activity, as indicated by reduced alpha power. Error bars represent Fisher’s least significant difference. *Statistically significant.

Analysis of the hemispheric alpha activity resulted in an interaction effect between Stimulation and Hemisphere (*F*_(1,7)_ = 9.75, *p* = 0.017, $\eta_{\text{G}}^{2}$ = 0.05). *Post hoc* analysis resulted in a main effect of Stimulation on the right hemisphere (*F*_(1,7)_ = 6.58, *p* = 0.037, $\eta_{\text{G}}^{2}$ = 0.3) but not the left hemisphere (*F*_(1,7)_ = 0.42, *p* = 0.54, $\eta_{\text{G}}^{2}$ = 0.03; Figure 3).

Reaction time analysis resulted in an interaction effect between Emotion and Stimulation (*F*_(1,7)_ = 20.75, *p* = 0.003, $\eta_{\text{G}}^{2}$ = 0.03). *Post hoc* analysis indicated a main effect of Emotion when ANT was stimulated (*F*_(1,7)_ = 16.66, *p* = 0.005, $\eta_{\text{G}}^{2}$ = 0.026), with slower reaction times in context of Emotional (512 ± 144 ms) than Neutral stimuli (495 ± 147 ms). There was also a main effect of Emotion when stimulation was OFF (*F*_(1,7)_ = 19.41, *p* = 0.003, $\eta_{\text{G}}^{2}$ = 0.22) with faster reaction times in context of Emotional (486 ± 118 ms) in contrast to Neutral stimuli (492 ± 119 ms).

### Correlation between Frontal Alpha Asymmetry Scores and Emotion-Related Reaction Times

**Figure 4:**
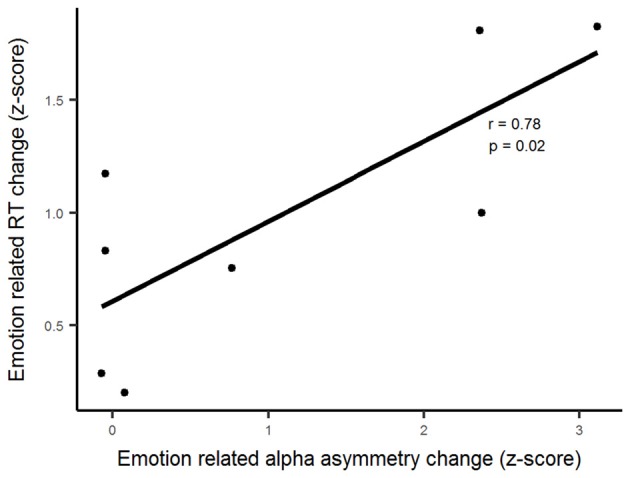
Deducted modulatory effects of emotion and ANT stimulation show a positive correlation between alpha asymmetry scores and the reaction times.

To investigate an association between the effect of ANT-DBS on emotional modulation of frontal alpha asymmetry and emotional modulation of RTs, we did a correlation analysis using Pearson’s correlation. The modulation of ANT-DBS on response speed and alpha asymmetry scores (see “Materials and Methods” section for the measures) linked to emotion were correlated (*r* = 0.69, *p* = 0.03; Figure 4).

## Discussion

We found in this study that during a cognitive task with emotional distractors high frequency electrical stimulation at ANT increased rightward frontal alpha asymmetry compared to stimulation at the thalamic control location or to no stimulation. Increased rightward frontal alpha asymmetry suggests relatively increased right frontal activity, which has been previously linked with anxiety disorders and depression (Coan and Allen, 2004; Thibodeau et al., 2006). This finding is in line with our previous finding of ANT-DBS increasing attention allocation to threat (Hartikainen et al., 2014; Sun et al., 2015), as well as with subjective reports of depression-related symptoms due to ANT-DBS (Fisher et al., 2010). Furthermore, we uncovered the effect of ANT-DBS on behavioral and physiological brain responses related to emotional stimuli with other contributing factors subtracted. After deriving these net ANT-DBS impact indices we found a correlation between emotion-linked alpha asymmetry scores and reaction times. We have previously shown that emotional modulation of frontal alpha asymmetry is also altered by another neuromodulation method impacting the affective circuits i.e., VNS (Sun et al., 2017). To that end, we suggest that task-related frontal alpha asymmetry along with measure of emotional interference of behavior could be used as a potential biomarker to objectively asses and gear the affective effects of neuromodulation treatments.

Hemispheric asymmetry in the prefrontal activation, as measured by the EEG power in the alpha band, has been proposed to predict brain’s response to affectively valenced stimuli (Davidson et al., 1990). Based on the Davidson’s approach-withdrawal theory, the left frontal brain region may be more active toward positive emotional stimuli linked with approach-related behaviors and the right frontal region more active toward negative emotional stimuli linked with withdrawal-related behaviors. Generally, alpha power decreases with increasing cognitive activity (Pfurtscheller et al., 1996; Klimesch, 1999). The frontal asymmetry is most commonly computed by subtracting the natural log of left hemisphere alpha power (EEG electrode F3) from the natural log of right hemisphere alpha power (F4) while using the central Cz as reference electrode (Coan and Allen, 2004). This approach generates a unidimensional scale indicating the relative activity of each brain hemisphere, with high asymmetry score indicating relatively greater left frontal activation and lower scores representing relatively greater right frontal activation. This traditional approach of measuring frontal alpha asymmetry based on asymmetry scores bears several advantages. Firstly, it simplifies the data analysis process and makes its interpretation somewhat straightforward. Further, compared to separate hemisphere analysis, it enables proper control of individual differences, including the subject specific behavioral activation and individual differences in skull thickness leading to non-neurogenic differences in absolute power values (Harmon-Jones and Allen, 1997; Sutton and Davidson, 1997; Coan and Allen, 2003). Also, these asymmetry scores are reported to show higher internal consistency (Tomarken et al., 1992; Jones et al., 1997).

Frontal alpha asymmetry may be a more robust marker than ERPs in detecting the affective effects of ANT-DBS in epilepsy patients. ERP analysis requires high quality EEG with few artifacts and identifiable ERP components difficult to obtain in some patients with refractory epilepsy (Hartikainen et al., 2014; Sun et al., 2015). EEG signal from patients with epilepsy is often contaminated by epileptic discharges and other pathological EEG oscillations resulting in indistinguishable and widely variable ERP components making the use of ERP derived biomarkers less reliable. Moreover, obtaining reliable ERP biomarkers requires several repetitions and long testing periods inconvenient in a clinical setting. Thus, robust and quickly derivable biomarkers allowing for immediate, online assessment of the impact of neuromodulation on affective circuits are needed.

Task-elicited frontal alpha asymmetry during emotional challenge has been shown to be a more robust indicator of vulnerability to depression than resting state alpha asymmetry (Stewart et al., 2014). In the current study, the cognitive task engaging frontal networks was combined with emotional challenge i.e., emotional distractor. The computer based test of executive functions used in the current study mimics everyday challenges with demand for variety of cognitive processes, like attention and executive control simultaneously. To that end, the task-related frontal alpha asymmetry may better reflect brain’s affective functions in the context of everyday cognitive challenges than resting state alpha asymmetry. Furthermore, combining EEG asymmetry measure with a behavioral task including measure of the extent of emotional interference allows for electrophysiological biomarker to be combined with a behavioral biomarker linking the observed effect of neuromodulation on both brain physiology and behavior.

Our findings along with previous literature suggest that ANT-DBS may modulate affective functions towards potential vulnerability to depression, as it causes increased right hemispheric activity and an increased attention allocation to threat, a hallmark for depressive symptoms. However, caution is warranted when extrapolating the current findings to clinical setting, partially due to our experimental setting of stimulation parameters differing from those used clinically. In our study, we used continuous stimulation with bipolar mode. In the clinical treatment, monopolar and intermittent stimulation is typically used (e.g., 1-min ON and 5 min OFF). Furthermore, we did not evaluate depression-related symptoms in this study, thus no direct conclusions can be drawn on whether the observed rightward alpha asymmetry and increased emotional interference of behavior due ANT-DBS reflect vulnerability for depression. Taken together, bridging the immediate effects found in the current study to long-term outcomes as well as directly linking these immediate brain and behavioral responses of ANT-DBS to subjective mood related symptoms require further studies.

The current findings highlight the role of ANT in human emotional processing. Although ANT has long been thought critical for the processing of emotion, and known as a key node of the Papez circuit (Papez, 1937), direct evidence supporting ANT’s role in human affective brain functions is scarce (Hartikainen et al., 2014; Sun et al., 2015). High-frequency electric stimulation at ANT, thought to mimic a reversible lesion, provides a unique opportunity to study its role in emotion and attention. Furthermore, turning this key node of the limbic circuitry on and off by means of DBS when subjects are actively engaged in a cognitive task and intermittently distracted by biologically relevant threat related emotional stimuli, while EEG and performance are measured, provides invaluable insight into affective brain circuits and how their function is reflected in behavioral and brain responses in humans. An approach allowing online manipulation of affective circuits and measurement of causally linked alterations in affective responses is also invaluable in identifying and assessing potential biomarkers for the effect of neuromodulation on brain’s affective circuitry in general.

Lack of objective signs of neuromodulation on affective circuits calls for on-line biomarkers for monitoring and optimizing neuromodulation treatment. Unlike when treating movement disorders with DBS where one can objectively observe an immediate reduction of movement related symptoms, when treating psychiatric disorders, such as depression and obsessive-compulsive disorder with DBS, there are no obvious signs reflecting the desired effects on limbic circuits which could be used to guide parameter selection towards the optimal treatment effect.

In the current study, we demonstrated the modulatory effect of ANT-DBS on brain’s affective circuitry and highlighted a potential new online biomarker for the immediate effects of neuromodulation on brain dynamics, i.e., combining frontal alpha asymmetry and emotional interference of response speed. However, we did not link these measures with mood related symptoms. Thus, future studies are needed to assess the utility of frontal alpha asymmetry during a task engaging frontal networks as a biomarker, which possibly could be used for minimizing the affective side effects of ANT-DBS in the treatment of refractory epilepsy and for guiding neuromodulation treatments of psychiatric disorders linked with frontal dysfunction and asymmetry, such as depression and anxiety, towards the optimal treatment effect.

In conclusion, we showed immediate effects on frontal alpha asymmetry due to neuromodulation targeted at a key node of the limbic circuit. Further, we provided causal evidence that neuromodulation which impacts affective circuits, ANT-DBS, influences alpha asymmetry and the modulation of alpha asymmetry is linked with emotional modulation of behavior. Thus, we conclude that an index derived from alteration of frontal alpha asymmetry along with emotional interference of performance reflect the functioning of limbic circuits and the impact of neuromodulation on them. While clinical relevance of such an index requires further research, it provides an objective measure for wide range of scientific experiments that aim either at better understanding dynamics of affective circuits, their dysfunction or the impact of neuromodulation on them. Furthermore, stimulating the two closely located thalamic regions lead to distinct lateralization effects suggesting frontal alpha asymmetry might be used to provide additional information on the DBS electrode location.

## Author Contributions

LS contributed to data analysis and writing the article. JP was involved in statistical analysis and writing the article. KMH contributed to experimental design, supervision of data analysis and writing the article.

## Conflict of Interest Statement

The authors declare that the research was conducted in the absence of any commercial or financial relationships that could be construed as a potential conflict of interest.
